# Efficacy of personalized exercise program on physical function in elderly patients with rheumatoid arthritis at high risk for sarcopenia: study protocol for a randomized controlled trial

**DOI:** 10.1186/s12891-023-06185-4

**Published:** 2023-04-11

**Authors:** Akira Onishi, Mie Torii, Yu Hidaka, Ryuji Uozumi, Yohei Oshima, Hiroki Tanaka, Hideo Onizawa, Takayuki Fujii, Koichi Murata, Kosaku Murakami, Masao Tanaka, Shuichi Matsuda, Akio Morinobu, Hidenori Arai, Motomu Hashimoto

**Affiliations:** 1 Department of Advanced Medicine for Rheumatic Diseases, Graduate School of Medicine, Kyoto University, Kyoto, Japan; 2 Department of Human Health Sciences, Graduate School of Medicine, Kyoto University, Kyoto, Japan; 3 Department of Biomedical Statistics and Bioinformatics, Graduate School of Medicine, Kyoto University, Kyoto, Japan; 4 Department of Industrial Engineering and Economics, Tokyo Institute of Technology, Tokyo, Japan; 5 Rehabilitation Unit, Kyoto University Hospital, Kyoto, Japan; 6 Department of Orthopaedic Surgery, Graduate School of Medicine, Kyoto University, Kyoto, Japan; 7 Division of Clinical Immunology and Cancer Immunotherapy, Center for Cancer Immunotherapy and Immunobiology, Graduate School of Medicine, Kyoto University, Kyoto, Japan; 8 Department of Rheumatology and Clinical Immunology, Graduate School of Medicine, Kyoto University, Kyoto, Japan; 9 National Center for Geriatrics and Gerontology, Obu, Aichi Japan; 10 Department of Clinical Immunology, Graduate School of Medicine, Osaka Metropolitan University, 1-4-3-13F, Asahi-cho, Abeno-ku, Osaka, Osaka 545-8585 Japan

**Keywords:** Rheumatoid arthritis, Sarcopenia, Exercise, Randomized controlled trial

## Abstract

**Background:**

Patients with rheumatoid arthritis (RA) are prone to muscle atrophy due to inflammatory cytokines and corticosteroid use and immobility due to joint pain and deformity. Although resistance training is effective and safe in reversing muscle atrophy in RA, some patients are unable to perform a conventional high-load exercise program due to disease-related limitations. This study aims to examine the efficacy of individualized exercise therapy on physical function in elderly patients with RA who are at a high risk for sarcopenia.

**Methods:**

This study is a single-center, parallel-group, two-arm, healthcare provider- and outcome assessor-blinded, superiority randomized controlled trial with a 1:1 allocation ratio. A total of 160 participants with RA between 60 and 85 years of age with a positive screening test for sarcopenia will be included. The intervention group will receive nutritional guidance and a four-month individualized exercise program in addition to the usual treatment. The control group will receive nutritional guidance in addition to the usual care. The primary endpoint will be physical function assessed using the Short Physical Performance Battery (SPPB) at 4 months. The data on outcome measures will be collected at baseline and at the two- and four-month follow-ups. Linear mixed-effects models for repeated measures will be conducted using the modified intention-to-treat analysis population.

**Discussion:**

This study will provide evidence on whether a personalized exercise program can improve physical function and quality of life in elderly patients with RA. Some limitations include limited generalizability due to the single-center study and lack of blinding of the patients to the intervention assignment because of the nature of the exercise. Physical therapists may use this knowledge in their daily practice to improve RA treatment. Tailored exercise may enhance the health outcomes of the RA population and contribute to a reduction in healthcare costs.

**Trial registration:**

The study protocol was retrospectively registered at the University hospital Medical Information Network-Clinical Trial Repository (UMIN-CTR) (registration number: UMIN000044930, https://www.umin.ac.jp/ctr/index-j.htm) on January 4, 2022.

**Supplementary Information:**

The online version contains supplementary material available at 10.1186/s12891-023-06185-4.

## Background

Patients with rheumatoid arthritis (RA) are prone to muscle atrophy due to inflammatory cytokines such as tumor necrosis factor α (TNFα) and interleukin 6 (IL-6). Corticosteroids, which are often used in the treatment of RA, also cause muscle atrophy owing to corticosteroid-induced myopathy. In addition, immobility due to joint pain and deformity leads to a lack of exercise, resulting in a vicious cycle of further decline in muscle strength and mass and physical function. Sarcopenia is a condition characterized by a steady reduction in skeletal muscle mass and strength and is associated with poor physical performance, functional impairment, and disability, resulting in high mortality and significant healthcare costs. In a previous study on sarcopenia in patients with RA, > 40% of patients with RA of ≥65 years of age had sarcopenia, which was significantly higher than that reported among the general healthy elderly population (5–17%) [1]. No specific drug therapy for sarcopenia has been established; since inflammatory cytokines such as TNFα and IL-6 produce muscle atrophy, biological agents such as TNF and IL-6 inhibitors used to treat RA may improve sarcopenia in patients with RA, but the evidence is currently limited.

Resistance training is effective and safe for reversing muscle atrophy in RA [2] whereas dietary protein supplementation and the combination with resistance exercise have shown an increase in muscle strength and physical performance in healthy elderly patients, but have not been completely examined in patients with RA [3, 4]. However, some patients are unable to perform conventional high-load exercise programs because of disease-related limitations [5]. In particular, exercise therapy has been reported to increase joint destruction in patients with advanced RA by inducing stress in the joints [6]; therefore, it is necessary to design novel and feasible exercise therapy tailored to individual patients with RA.

## Methods/design

### Trial design

This study aims to examine the efficacy of individualized exercise therapy on physical function in elderly patients with RA who are at a high risk for sarcopenia. This study is a single-center, parallel-group, two-arm, healthcare provider-and outcome assessor-blinded, superiority randomized controlled trial with a 1:1 allocation ratio (trial registration number: UMIN000044930). This study will be conducted at the Rheumatology Center, Kyoto University Hospital, Kyoto, Japan. The study protocol follows the Standard Protocol Items: Recommendations for Interventional Trials (SPIRIT) (Additional file 1) [7, 8]. The report will be presented as a Consolidated Standards of Reporting Trials (CONSORT) Statement [9, 10]. This study was approved by the ethics committee of Kyoto University (approval number: C1534–1). This study will be conducted in accordance with the principles of the Declaration of Helsinki. Written informed consent will be obtained from all participants by the attending physicians. Participants could withdraw from the trial at any time. On the consent form, participants will be asked if they agree that their data can be used should they choose to withdraw from the trial. This trial will involve collecting biological specimens for storage. The protocol was initially released on December 15, 2021, and updated as version 6.3 on October 4, 2022. No significant modifications to the protocol are expected. However, if any changes are required, they will have to be reviewed by the ethics committee of Kyoto University, Kyoto, Japan. The results of this trial will be presented at national or international conferences, and papers will be submitted to peer-reviewed journals.

Participants will be recruited from the outpatient rheumatology clinics at Kyoto University Hospital. To achieve adequate participant enrollment to reach the target sample size and promote participant retention and complete follow-up, incentives will be given as gift certificates worth 1000 yen (3000 yen in total) at the initial evaluation (month 0), first post-assignment evaluation (month 2), and second post-assignment evaluation (month 4) for both intervention and non-intervention groups.

The trial is ongoing and is currently recruiting patients. Recruitment was initiated on January 20th, 2022 and is expected to be completed by the end of July 2023.

### Eligibility criteria

Participants with RA who meet all the inclusion criteria and none of the exclusion criteria will be enrolled in the study. The inclusion criteria are as follows: 1) age between 60 and 85 years at enrollment, 2) diagnosed with RA according to the 2010 American College of Rheumatology/European League Against Rheumatism classification criteria for RA [11], and 3) positive screening test for sarcopenia (hand-grip strength: < 28 kg for men and < 18 kg for women; or five times chair-stand test ≥12 s) [12]. The exclusion criteria are: 1) participants who have difficulty in walking or standing up without any assistance; 2) patients who are instructed by their physician to limit their exercise; 3) patients with a pacemaker; 4) patients with cognitive impairment (Mini-Mental State Examination score ≤ 23) [13]; and 5) participants deemed inappropriate for this study by investigators.

### Trial procedures

The intervention group will receive nutritional guidance and a four-month individualized exercise program in addition to the usual treatment. The control group will receive nutritional guidance in addition to the usual care. The individualized exercise program will be developed by physical therapists by modifying previous exercise programs [14–16]. The program will be individualized according to the muscle strength and physical functions of the patients and can be conducted at home. The program will be offered for 4 months and will include resistance and aerobic exercises. Resistance exercise will consist of six types for the lower limbs and two types for the hands, and will be conducted for approximately 30–60 minutes per time at three non-consecutive times per week. The details of the resistance exercise are shown in the Supplementary materials. The exercise will be adjusted monthly according to the Borg scale, a scale to measure the perceived physical activity intensity level [17]. Lower limb exercises will begin at level 1. The starting level of hand exercises will be determined according to the Borg scale for squeezing a ball. Subjective exercise intensity will be set at 5 to 6 (hard) using the Borg scale, so that the load is 50–70% of the maximum muscle strength. The aerobic exercise program will encourage patients to achieve the target number of steps provided by the investigators. The target number of steps between baseline and 2 months will be determined as a 20% increase in the number of walks from baseline. The target number of steps between two and 4 months will be determined as a 20% increase in the number of walks at 2 months. The exercise program will be implemented as follows: 1) research nurses trained by physical therapists will instruct the exercise program to patients, 2) patients will perform the instructed exercise program at home at a pre-determined frequency and record the exercises performed, and 3) when the patient visits the clinic every month during the study period, the adjusted exercise program will be provided according to record sheets.

The criteria for discontinuing allocated interventions include: 1) difficulty in continuing the study due to the development or worsening of comorbidities, 2) participant requests for discontinuation of treatment or withdrawal of consent, 3) failure to meet the eligibility criteria found after the start of treatment, 4) inability to visit the hospital due to relocation or contact with participants, 5) participant’s death, and 6) difficulty in continuing the study by the principal investigator or sub-investigators. The date and timing of discontinuation (treatment period and follow-up period) and the reason for discontinuation will be recorded, and at the time of discontinuation, the efficacy and safety will be evaluated in terms of primary and secondary endpoints.

To assess the quality of the exercise program conducted by the participants, they will be asked to show their exercise content twice at each visit to the research nurses. In addition, the performance of participants who gave their consent will be recorded, and randomly selected recorded data will be evaluated for the quality of the exercise program by supervising physical therapists. To evaluate adherence to the exercise program, patients will be asked to record the details of the exercise conducted and the Borg scale. In addition, the patients will be asked to wear an activity meter once every 2 months, and the activity measured with the meter will be checked against the exercise records to confirm the degree of adherence.

Nutritional guidance will include an explanation of nutrients, such as proteins, which are generally considered good for preventing sarcopenia, and recipes that can be easily prepared by individuals. This content will be developed by nutritionists, and guidance will be provided by research nurses under the guidance of nutritionists.

Any changes in usual care will be permitted at the discretion of the attending physician. However, the details of the usual treatment at baseline and at each subsequent visit will be recorded. Post-trial care will not be provided.

### Trial outcomes

The primary endpoint will be the Short Physical Performance Battery (SPPB) at 4 months [18]. The SPPB is a 12-point scale with higher scores indicating better physical function and consists of balance, gait speed, and chair-stand tests. Diagnostic criteria for sarcopenia were proposed by the Asian Working Group for Sarcopenia (AWGS), which consists of physical function, skeletal muscle mass, and grip strength; however, biomarkers to measure sarcopenia have not been identified [12]. The main objective of this study is to improve physical function measured by the SPPB. Since previous studies have shown that grip strength and skeletal muscle mass were not expected to change in the short-to-medium term compared to physical function, they will be examined as secondary endpoints.

The secondary endpoints will be measured at 4 months and include each component of the SPPB (balance, gait speed, and chair-stand tests); grip strength; limb skeletal muscle index; physical activity measured with an activity meter and International Physical Activity Questionnaire (IPAQ) [19, 20]; disease activity of RA measured with Disease Activity Score 28 joints (DAS28) [21], Clinical Disease Activity Index (CDAI) [22], and Simplified Disease Activity Index (SDAI) [23]; disability level measured with Health Assessment Questionnaire (HAQ) [24]; overall health-related quality of life measured using the EuroQol-5 Dimension-3 Level (EQ-5D-3L) [25] and 12-Item Short-Form Health Survey (SF-12) [26, 27]; and subjective depression and anxiety symptoms measured with Hospital Anxiety and Depression Scale (HADS) [28, 29]. Adverse events that may be related to this study (such as pain, fall, fracture, orthopedic hypotension, headache, and dizziness) will be recorded at each outpatient visit. Adverse events that may occur during the trial period will be self-reported by patients. Researchers supervising the interventions and collecting outcome measures at follow-up appointments will also identify possible adverse events.

### Timeline of assessments

**Fig. 1:**
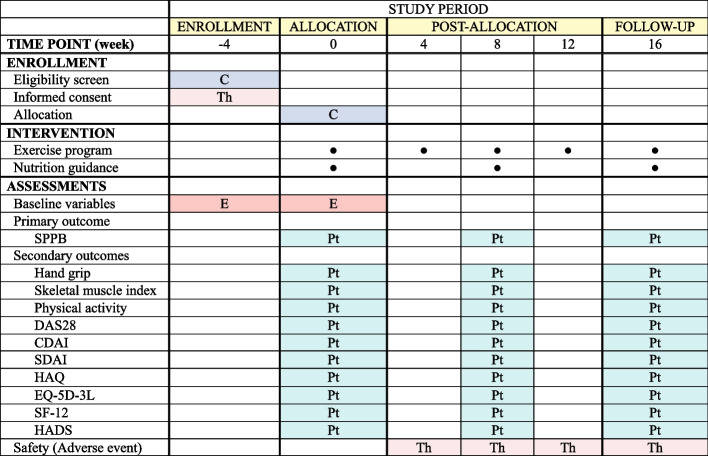
Time schedule of enrollment, allocation, interventions, assessments, and visits to participants C: Coordinator, E: Evaluator, Pt: Patient, Th: Therapist. CDAI: Clinical Disease Activity Index; DAS28: Disease Activity Score 28 joints; EQ-5D-3L: EuroQol-5 Dimension-3 Level; HADS: Hospital Anxiety and Depression Scale; HAQ: Health Assessment Questionnaire; SDAI: Simplified Disease Activity Index; SF-12: 12-Item Short-Form Health Survey; SPPB: Short Physical Performance Battery

Baseline assessments will be conducted after informed consent has been obtained. In addition to the outcomes, the collected data will include participant age, sex, height, weight, body mass index (BMI), marital status, education level, occupation, income level, smoking, alcohol consumption status, disease duration, medical history of RA, comorbidity, past medical history, and surgical history including joint replacement surgery. The schedule of enrollment, allocation, interventions, assessments, and visits for the participants is shown in Fig. 1.

### Randomization and blinding

Patients will be centrally randomized to the intervention and control groups after enrollment and nutritional guidance according to computer-generated random numbers created by the coordinating center. By using permuted block randomization stratified according to sex and BMI, participants will be randomly assigned to 2 × 2 strata of sex (male and female) and BMI (≥25 and < 25) with an allocation ratio of 1:1 for each stratum.

The healthcare providers and outcome assessors will be blinded; however, it is not possible to blind participants due to the nature of the exercise program interventions. To assess the achievement of blinding of healthcare providers, a questionnaire on knowledge of intervention will be administered after a four-month exercise program. The commencement of the intervention will be defined as the start of the individualized exercise program after the assignment, and the completion of the intervention will be defined as the completion of the measurement after a four-month exercise program.

### Data management and monitoring

Information on the eligibility criteria will be collected on paper via telephone calls from the central office. Baseline information and outcome assessment during the study will be completed by the participants and evaluators, and data will be entered by the study personnel. The data on changes in medications will also be collected on paper by the attending physicians. Adverse events that may occur during the trial period will be self-reported by patients. Attending physicians and researchers collecting data on outcome measures at follow-up appointments will also identify possible adverse events. Any missing values will be queried on the patients’ visits or by telephone.

The principal investigator will be responsible for the secure storage of the participant data. The study data will be anonymized by assigning a study number to each participant and archived in a password-protected database on secure servers. The study data will only be accessible to the investigators. Auditing will not be conducted because the intervention in this study is minimally invasive.

### Statistical analysis

Based on the results from previous studies [30–32], we estimated that 128 patients would provide the trial with 80% statistical power at a two-sided significance level of 0.05 to detect a difference of 1 point between the intervention and control groups with a standard deviation of 2 points in the SPPB score at 4 months (primary outcome). We plan to enroll 160 patients to allow 20% of the patients to withdraw from the trial.

Baseline characteristics will be presented as mean and standard deviation for continuous variables and as count and percentage for categorical variables. The primary analysis will be conducted using the intention-to-treat population. A linear mixed-effects model for repeated measures will be used to estimate the change in the SPPB score from baseline to 4 months. The fixed effects in the model will include SBBP score at baseline, trial group, visit, interaction of trial group with visit, and age at baseline. Mixed-effects models for repeated measures will also be considered for secondary outcomes. Subgroup analyses will be conducted based on the duration of RA, use of biologics and Janus kinase inhibitors, and remission of disease activity. The number and percentage of patients with adverse events will be calculated for each group. For the primary outcome, a two-tailed *P* value of < 0.05 will be considered to indicate statistical significance. For all other outcomes, point estimates and 95% confidence intervals will be reported. The widths of confidence intervals will not be adjusted for multiplicity. There is no plan for any interim analysis or early stopping. Analyses will be performed using Stata software, version 17 (StataCorp, College Station, TX), SAS software, version 9.4 (SAS Institute, Cary, NC), or R software, version 4.10 (R Foundation for Statistical Computing, Vienna, Austria).

## Discussion

To the best of our knowledge, this is the largest randomized controlled trial to examine the efficacy of a personalized exercise program on physical function in elderly patients with RA who are at a high risk for sarcopenia. Resistance training is considered as an effective exercise intervention to improve muscle strength and counteract muscle loss in RA [2]. Although the American College of Sports Medicine recommends that training loads corresponding to 60–70% of a repetition maximum are necessary to promote muscle strength and mass accrual [33], conventional high-load resistance training may not be feasible in all RA patients because of pain, fatigue, and joint limitations imposed by the disease [5]. A previous study showed that 82% of patients in the intensive exercise program had lower resistance or less exercise time than planned because of pain or fatigue. Similarly, 65% of the patients adjusted the exercise load for the resistance exercises [5]. Therefore, the development of novel and feasible strategies to counteract RA-related muscle mass and strength loss is necessary. When a tailored exercise program improves physical function, the results of this study will offer a more feasible alternative to uniform resistance training for treating patients with more severe joint disease.

The current study has several limitations. First, because this is a single-center study that mainly consists of stable established RA, the generalizability to active early RA is limited. Second, although exercise can be individualized to improve the feasibility of the exercise program, the average effect of the individualized exercise program may depend on the characteristics of the study population such as age, muscle mass, pain, joint destruction, and disease activity. Therefore, we will conduct several subgroup analyses to assess the effect modification of the exercise programs. Third, the patients will not be blinded to the intervention assignment because blinding would be difficult considering the nature of the intervention. In contrast, the physician and outcome assessor will be blinded. Therefore, objective outcomes may be minimally biased, whereas subjective outcomes may be affected by information bias.

This study will provide evidence as to whether a personalized exercise program can improve physical function and quality of life in elderly patients with RA. Physical therapists may use this knowledge in their daily practice to improve RA treatment. Tailored exercise may enhance the health outcomes of the RA population and contribute to a reduction in healthcare costs. Considering that the key elements that constitute an individual exercise program are easily applicable, this will, in turn, infer the potential translation to other inflammatory conditions.

## Supplementary Information

**Additional file 1.** SPIRIT-Outcomes 2022 Checklist (for combined completion of SPIRIT 2013 and SPIRITOutcomes 2022 items)^a^

**Additional file 2.**

## Data Availability

Not applicable.

## Abbreviations

BMI: Body mass index
CDAI: Clinical Disease Activity Index
CONSORT: Consolidated Standards of Reporting Trials
DAS28: Disease Activity Score 28 joints
EQ-5D-3L: EuroQol-5 Dimension-3 Level
HADS: Hospital Anxiety and Depression Scale
HAQ: Health Assessment Questionnaire
IL-6: Interleukin 6
IPAQ: International Physical Activity Questionnaire
RA: Rheumatoid arthritis
SDAI: Simplified Disease Activity Index
SF-12: 12-Item Short-Form Health Survey
SPPB: Short Physical Performance Battery
SPRINT: Standard Protocol Items: Recommendations for Interventional Trials
TNF: Tumor necrosis factor
