# Supplementary material for: Efficacy of personalized exercise program on physical function in elderly patients with rheumatoid arthritis at high risk for sarcopenia: study protocol for a randomized controlled trial
Source: BMC Musculoskelet Disord. 2023 Apr 11;24:280. doi: 10.1186/s12891-023-06185-4 (PMC10088120; doi:10.1186/s12891-023-06185-4)
Supplement: Supplementary file 2 — Additional file 2. [file 12891_2023_6185_MOESM2_ESM.pptx]

## Slide 1
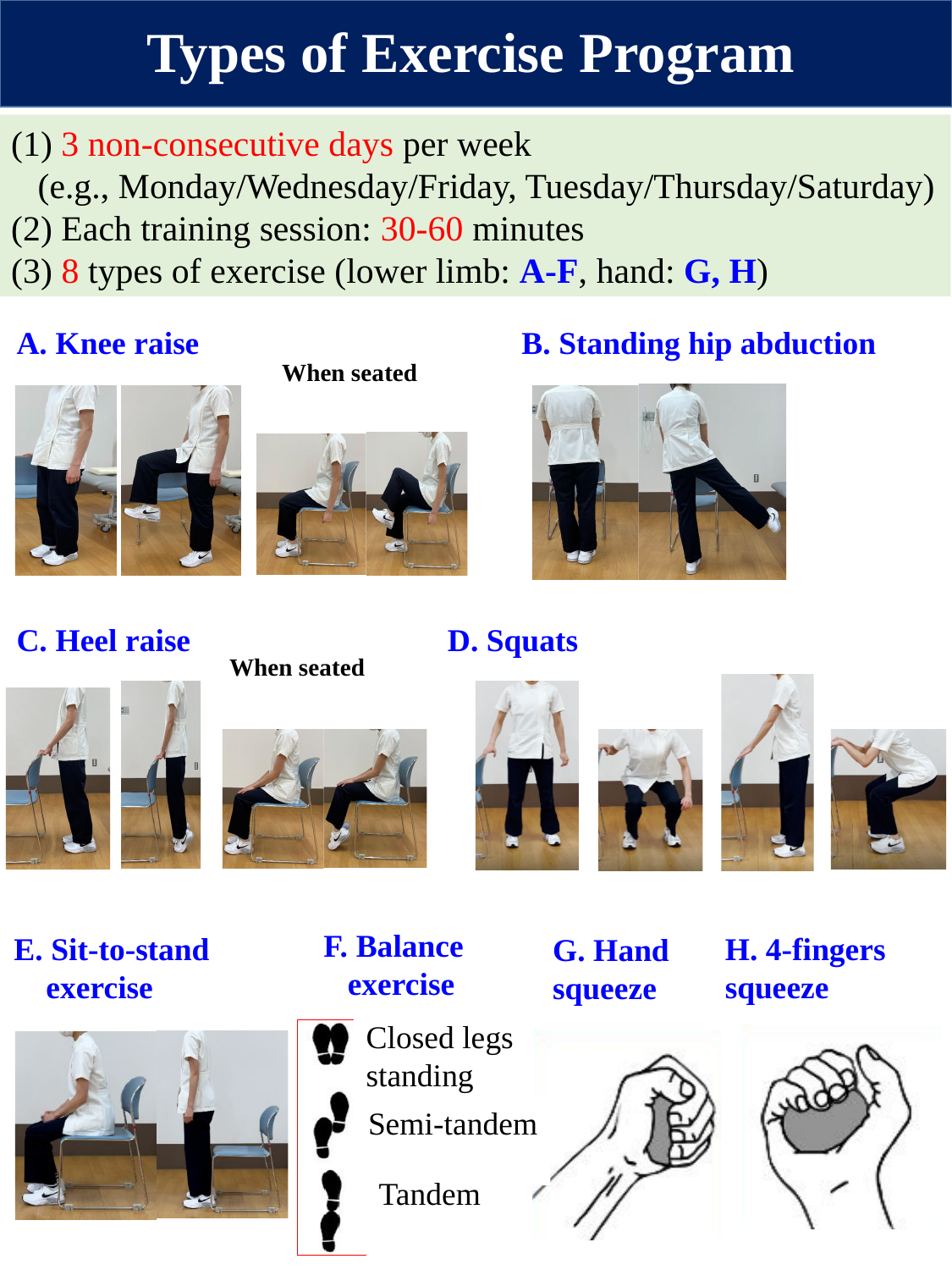

Types of Exercise Program
(1) 3 non-consecutive days per week
 (e.g., Monday/Wednesday/Friday, Tuesday/Thursday/Saturday)
(2) Each training session: 30-60 minutes
(3) 8 types of exercise (lower limb: A-F, hand: G, H)
A. Knee raise
B. Standing hip abduction
When seated
D. Squats
C. Heel raise
When seated
F. Balance
 exercise
E. Sit-to-stand
 exercise
H. 4-fingers squeeze
G. Hand squeeze
Closed legs
standing
Semi-tandem
Tandem

## Slide 2
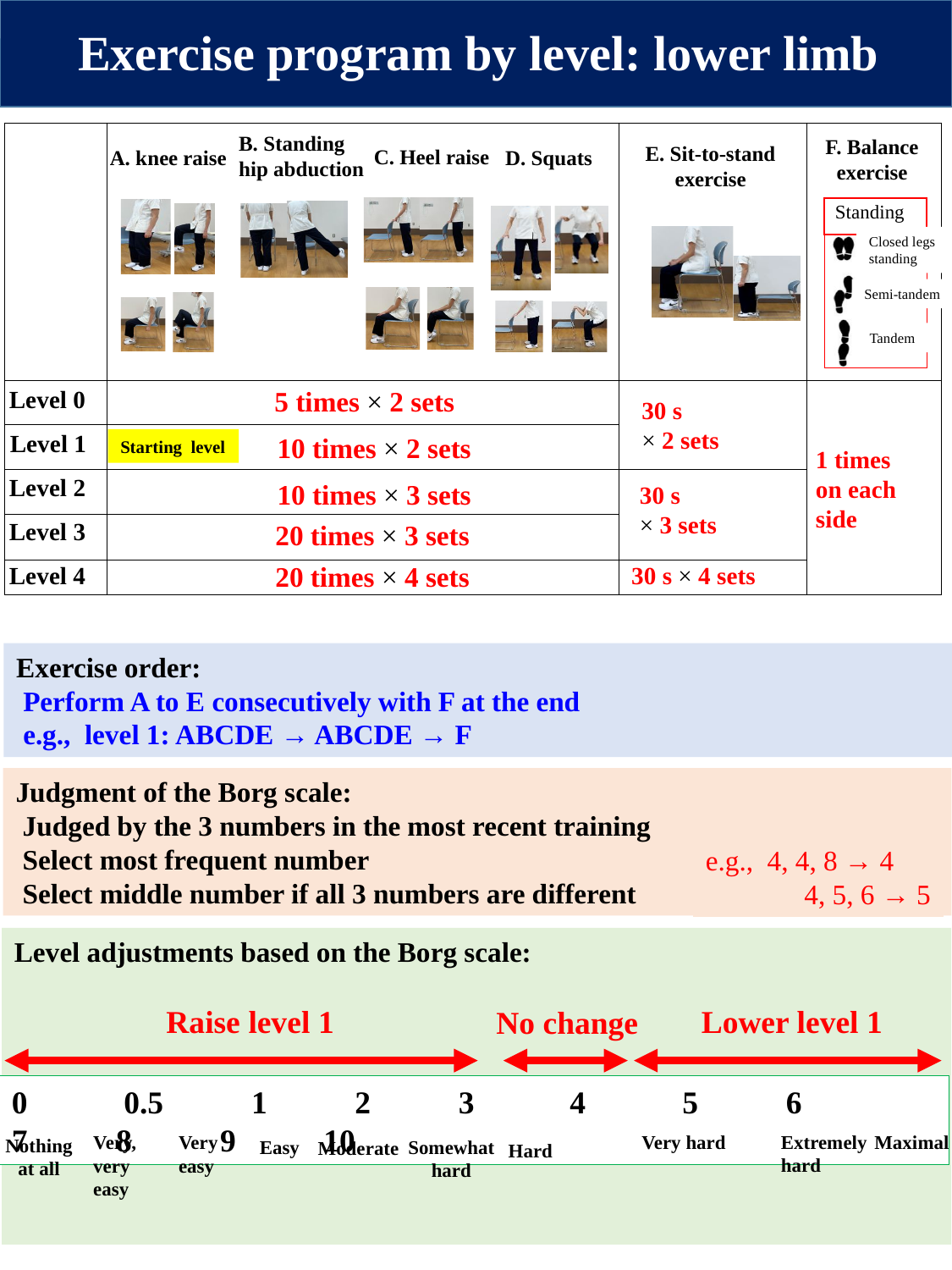

Exercise program by level: lower limb
| | | | |
| --- | --- | --- | --- |
| | | | |
| | | | |
| | | | |
| | | | |
| | | | |
B. Standing
hip abduction
F. Balance exercise
E. Sit-to-stand exercise
C. Heel raise
A. knee raise
D. Squats
Standing
Closed legs
standing
Semi-tandem
Tandem
5 times × 2 sets
Level 0
30 s
× 2 sets
Level 1
10 times × 2 sets
Starting level
1 times
on each side
Level 2
10 times × 3 sets
30 s
× 3 sets
Level 3
20 times × 3 sets
20 times × 4 sets
30 s × 4 sets
Level 4
Exercise order:
 Perform A to E consecutively with F at the end
 e.g., level 1: ABCDE → ABCDE → F
Judgment of the Borg scale:
 Judged by the 3 numbers in the most recent training
 Select most frequent number
 Select middle number if all 3 numbers are different
e.g., 4, 4, 8 → 4
　　　 4, 5, 6 → 5
Level adjustments based on the Borg scale:
Raise level 1
Lower level 1
No change
0 　 0.5 　 1 　 2 　 3 　 4　 5 　 6 　7　 8　 9 　 10
Extremely hard
Very, very easy
Very easy
Very hard
Maximal
Nothing at all
Easy
Somewhat hard
Moderate
Hard

## Slide 3
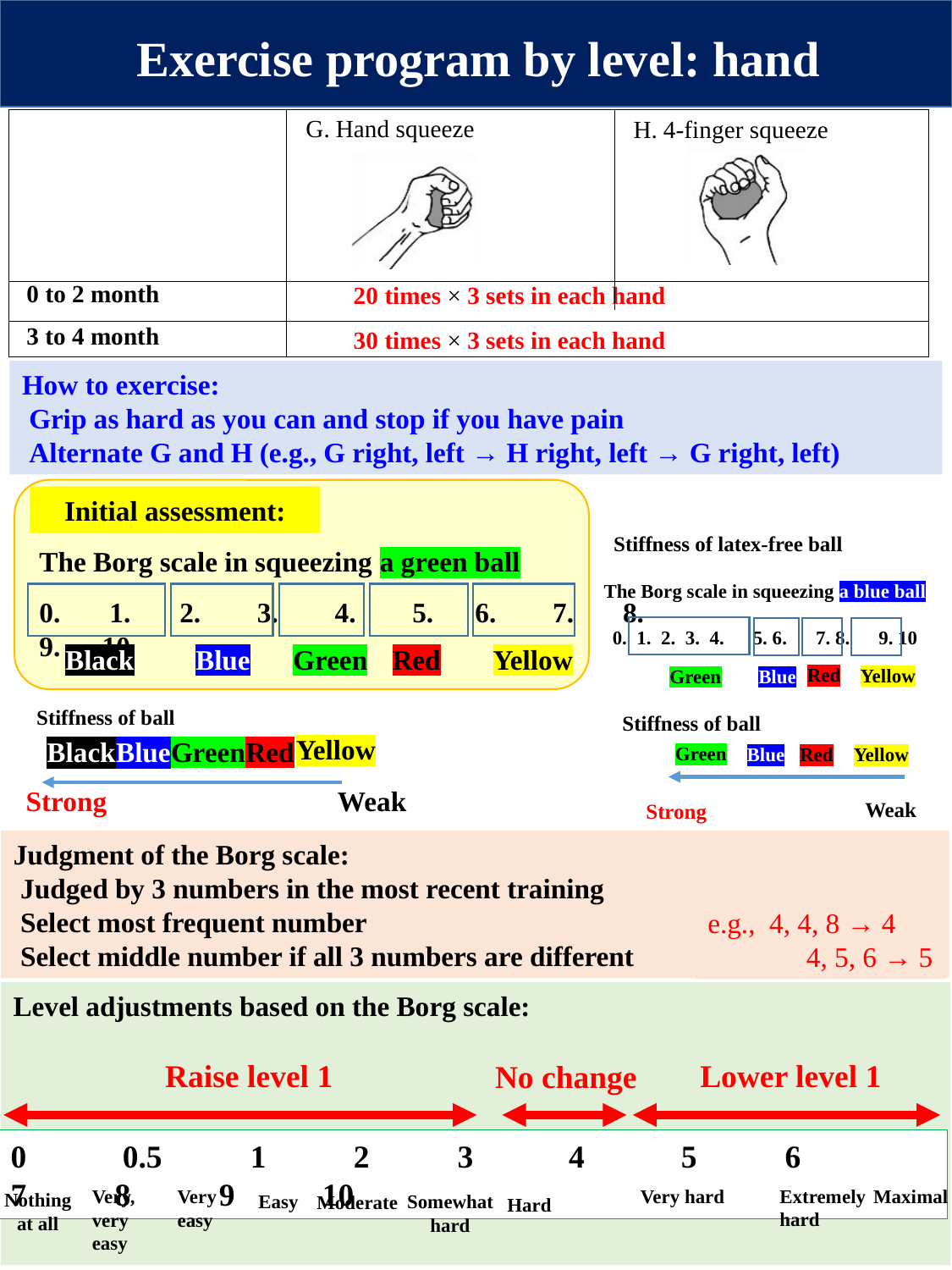

Exercise program by level: hand
G. Hand squeeze
H. 4-finger squeeze
| | |
| --- | --- |
| | |
| | |
0 to 2 month
20 times × 3 sets in each hand
3 to 4 month
30 times × 3 sets in each hand
How to exercise:
 Grip as hard as you can and stop if you have pain
 Alternate G and H (e.g., G right, left → H right, left → G right, left)
Initial assessment:
Stiffness of latex-free ball
The Borg scale in squeezing a green ball
0.　 1.　 2.　 3.　 4.　 5.　6.　 7.　 8.　 9.　10
The Borg scale in squeezing a blue ball
0. 1. 2. 3. 4.　5. 6.　7. 8.　9. 10
Black
Blue
Green
Red
Yellow
Red
Yellow
Green
Blue
Stiffness of ball
Stiffness of ball
Yellow
Green
Red
Blue
Black
Green
Blue
Red
Yellow
Strong
Weak
Weak
Strong
Judgment of the Borg scale:
 Judged by 3 numbers in the most recent training
 Select most frequent number
 Select middle number if all 3 numbers are different
e.g., 4, 4, 8 → 4
　　　 4, 5, 6 → 5
Level adjustments based on the Borg scale:
Raise level 1
Lower level 1
No change
0 　 0.5 　 1 　 2 　 3 　 4　 5 　 6 　7　 8　 9 　 10
Extremely hard
Very, very easy
Very easy
Very hard
Maximal
Nothing at all
Easy
Somewhat hard
Moderate
Hard

## Slide 4
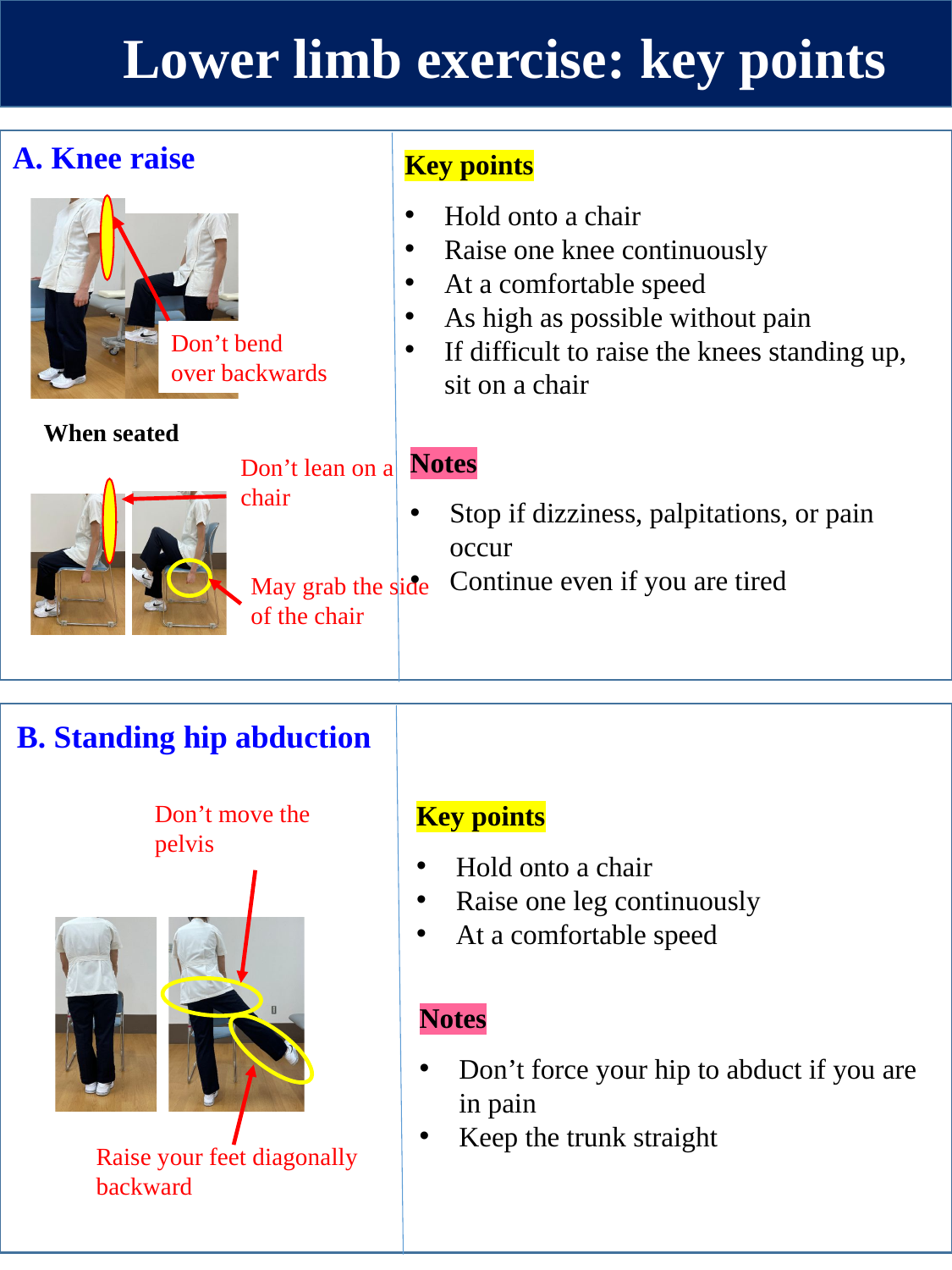

Lower limb exercise: key points
A. Knee raise
Key points
Hold onto a chair
Raise one knee continuously
At a comfortable speed
As high as possible without pain
If difficult to raise the knees standing up, sit on a chair
Don’t bend
over backwards
When seated
Notes
Stop if dizziness, palpitations, or pain occur
Continue even if you are tired
Don’t lean on a chair
May grab the side of the chair
B. Standing hip abduction
Don’t move the pelvis
Key points
Hold onto a chair
Raise one leg continuously
At a comfortable speed
Notes
Don’t force your hip to abduct if you are in pain
Keep the trunk straight
Raise your feet diagonally backward

## Slide 5
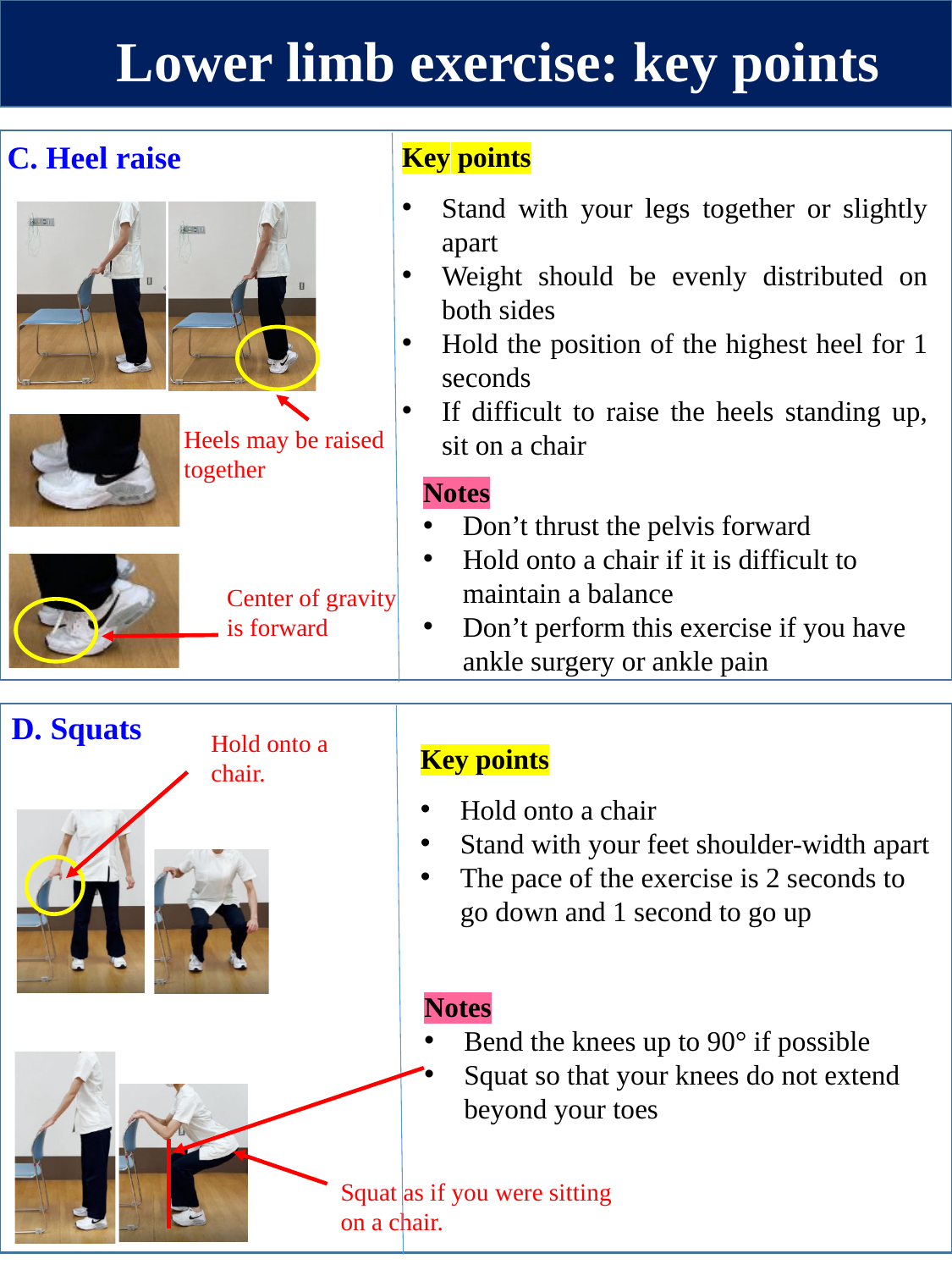

Lower limb exercise: key points
C. Heel raise
Key points
Stand with your legs together or slightly apart
Weight should be evenly distributed on both sides
Hold the position of the highest heel for 1 seconds
If difficult to raise the heels standing up, sit on a chair
Heels may be raised together
Notes
Don’t thrust the pelvis forward
Hold onto a chair if it is difficult to maintain a balance
Don’t perform this exercise if you have ankle surgery or ankle pain
Center of gravity is forward
D. Squats
Hold onto a chair.
Key points
Hold onto a chair
Stand with your feet shoulder-width apart
The pace of the exercise is 2 seconds to go down and 1 second to go up
Notes
Bend the knees up to 90° if possible
Squat so that your knees do not extend beyond your toes
Squat as if you were sitting on a chair.

## Slide 6
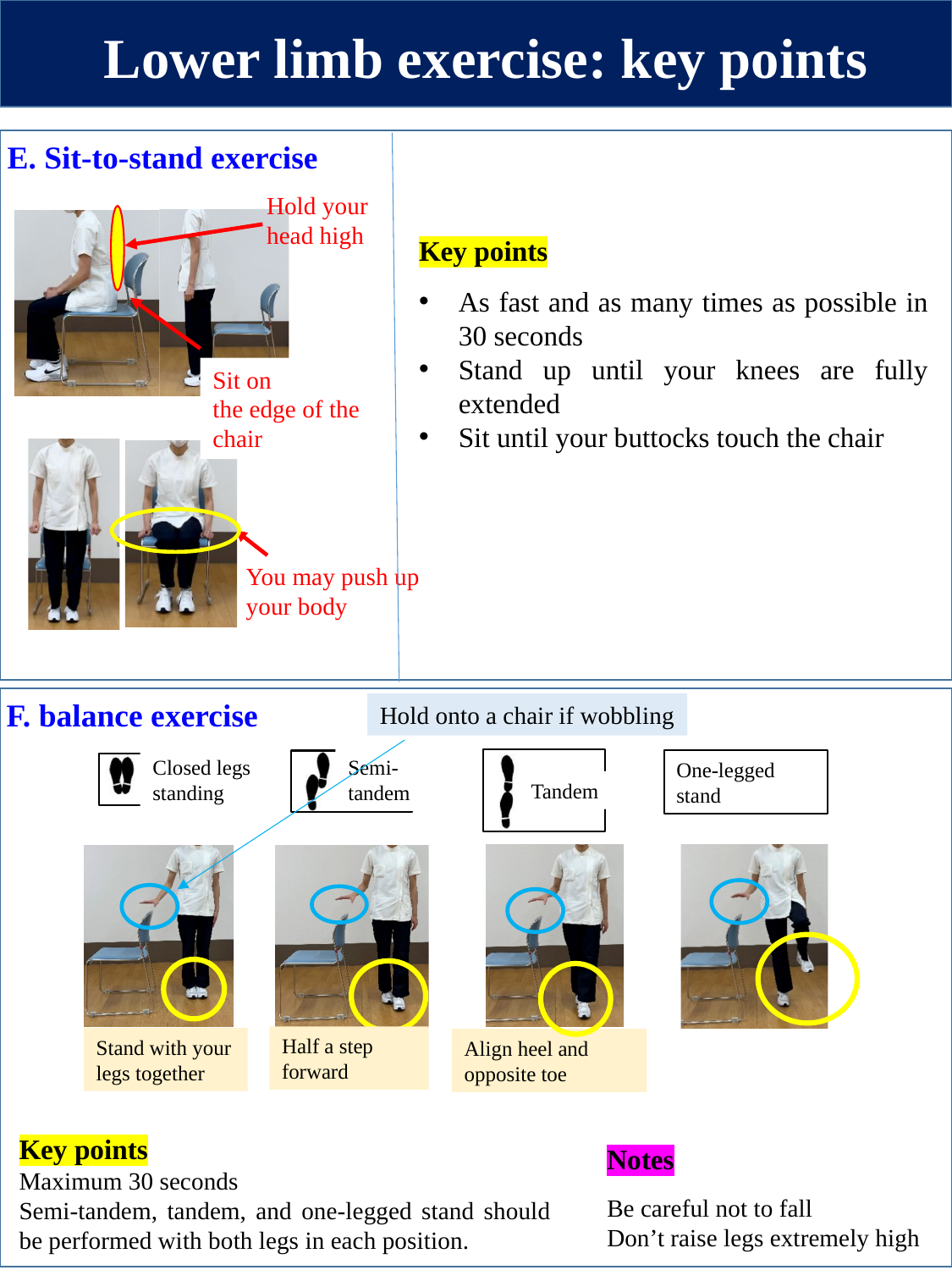

Lower limb exercise: key points
E. Sit-to-stand exercise
Hold your
head high
Key points
As fast and as many times as possible in 30 seconds
Stand up until your knees are fully extended
Sit until your buttocks touch the chair
Sit on
the edge of the chair
You may push up your body
F. balance exercise
Hold onto a chair if wobbling
Closed legs
standing
Semi-tandem
One-legged stand
Tandem
Half a step forward
Stand with your legs together
Align heel and opposite toe
Key points
Maximum 30 seconds
Semi-tandem, tandem, and one-legged stand should be performed with both legs in each position.
Notes
Be careful not to fall
Don’t raise legs extremely high
